# Effect of a high dose atorvastatin as added-on therapy on symptoms and serum AMPK/NLRP3 inflammasome and IL-6/STAT3 axes in patients with major depressive disorder: randomized controlled clinical study

**DOI:** 10.3389/fphar.2024.1381523

**Published:** 2024-05-24

**Authors:** Khlood Mohammad Aldossary, Lashin Saad Ali, Mahmoud S. Abdallah, Mostafa M. Bahaa, Thanaa A. Elmasry, Eman I. Elberri, Fedaa A. Kotkata, Ramy M. El Sabaa, Yasmine M. Elmorsi, Mostafa M. Kamel, Walaa A. Negm, Aya Ibrahim Elberri, Amir O. Hamouda, Hayam Ali AlRasheed, Muhammed M. Salahuddin, Mohamed Yasser, Manal A. Hamouda

**Affiliations:** ^1^ Department of Pharmacy Practice, College of Pharmacy, Princess Nourah bint Abdulrahman University, Riyadh, Saudi Arabia; ^2^ Department of Basic Medical Science, Faculty of Dentistry, Al-Ahliyya Amman University, Amman, Jordan; ^3^ Physiology Department, Faculty of Medicine, Mansoura University, Mansoura, Egypt; ^4^ Department of Clinical Pharmacy, Faculty of Pharmacy, University of Sadat City (USC), Sadat City, Menoufia, Egypt; ^5^ Department of PharmD, Faculty of Pharmacy, Jadara University, Irbid, Jordan; ^6^ Pharmacy Practice Department, Faculty of Pharmacy, Horus University, New Damietta, Egypt; ^7^ Pharmacology and Toxicology Department, Faculty of Pharmacy, Tanta University, Tanta, Al-Gharbia, Egypt; ^8^ Department of Clinical Pharmacy, Faculty of Pharmacy, Tanta University, Tanta, Al-Gharbia, Egypt; ^9^ Department of Clinical Pharmacy, Faculty of Pharmacy, Menoufia University, Shebin El-Kom, Menoufia, Egypt; ^10^ Psychiatry Department, Faculty of Medicine, Tanta University, Egypt; ^11^ Pharmacognosy Department, Faculty of Pharmacy, Tanta University, Tanta, Al-Gharbia, Egypt; ^12^ Genetic Engineering and Molecular Biology Division, Department of Zoology, Faculty of Science, Menoufia University, Shebin El-Kom, Menoufia, Egypt; ^13^ Department of Biochemistry and Pharmacology, Faculty of Pharmacy, Horus University, New Damietta, Egypt; ^14^ Department of Pharmaceutics, Faculty of Pharmacy, Port Said University, Port Said, Egypt; ^15^ Department of Pharmaceutics and Industrial Pharmacy, Faculty of Pharmacy, Horus University, New Damietta, Egypt

**Keywords:** atorvastatin, major depressive disorder, neuroinflammation, NLRP-3, STAT-3

## Abstract

**Background:**

Neuroinflammation pathways have been associated with the development of major depressive disorders (MDD). The anti-inflammatory characteristics of statins have been demonstrated to have significance in the pathophysiology of depression.

**Aim:**

To investigate the mechanistic pathways of high dose atorvastatin in MDD.

**Patients and methods:**

This trial included 60 patients with MDD who met the eligibility requirements. Two groups of patients (n = 30) were recruited by selecting patients from the Psychiatry Department. Group 1 received 20 mg of fluoxetine plus a placebo once daily. Group 2 received fluoxetine and atorvastatin (80 mg) once daily. All patients were assessed by a psychiatrist using the Hamilton Depression Rating Scale (HDRS). A HDRS score of ≤7 indicates remission or partial remission [HDRS<17 and>7]. Response was defined as ≥ 50% drop in the HDRS score. The serum concentrations of nucleotide-binding domain, leucine-rich-containing family, pyrin domain-containing-3 (NLRP-3), interleukin-6 (IL-6), adenosine monophosphate activated protein kinase (AMPK), and signal transducer and activator of transcription factor-3 (STAT-3) were measured.

**Results:**

The atorvastatin group showed a significant reduction in the levels of all measured markers along with a statistical increase in the levels of AMPK when compared to the fluoxetine group. The atorvastatin group displayed a significant decrease in HDRS when compared to its baseline and the fluoxetine group. The response rate and partial remission were higher in the atorvastatin group than fluoxetine (*p* = 0.03, and *p* = 0.005), respectively.

**Conclusion:**

These results imply that atorvastatin at high doses may be a promising adjuvant therapy for MDD patients by altering the signaling pathways for AMPK/NLRP3 and IL-6/STAT-3.

**Clinical Trial Registration:**

clinicaltrials.gov, identifier NCT05792540.

## 1 Introduction

Major depressive disorder (MDD) affects approximately 16% of people worldwide, a major cause of disability that has been associated with ongoing inflammatory conditions (Wittenberg et al., 2020). Though the existing antidepressants are generally successful and focus on the monoamine and serotonin pathways, more than 30% of patients rarely fully recover (Wittenberg et al., 2020). There is evidence that the immunological and neurological systems are strongly correlated (Leonard, 2018). Inflammations, whether acute or chronic, can have a direct or indirect impact on how the central nervous system (CNS) works. Tumor necrosis factor alpha (TNF-α) and interleukin (IL)-6 are examples of proinflammatory mediators that can directly activate neutrophils and macrophages, leading to the generation of oxygen and nitrogen free radicals (Jo et al., 2015). Alternatively, cytokines have an indirect effect on the CNS by altering the monoaminergic systems that are the main target of FDA-approved antidepressant drugs. Thus, a rise in cytokine levels causes a reduction in serotonin levels by activating the metabolic pathways for competing tryptophan and by increasing its uptake (Jo et al., 2015). Additionally, it has been noted that MDD is mostly associated with an increase in these proinflammatory cytokines (Jo et al., 2015).

Although the causes of MDD are still unclear, the NLRP3 inflammasome has been linked to the development of depression (4). First, it has been discovered that rodent depression models and people with MDD both have active NLRP3 inflammasomes (Taene et al., 2020). Second, a functional NLRP3 inflammasome is necessary for depressed behaviors brought on by stress, while NLRP3 inhibition prevents these stress-like effects (Du and Bu, 2020). Third, antidepressant therapy may prevent the activation of the NLRP3 inflammasome (Du and Bu, 2020). These results postulated that the NLRP3 inflammasome might be a major target for novel depression therapeutic approaches. AMP-activated protein kinase (AMPK) is a ubiquitous fuel-sensing enzyme that has a vital role in the control of the metabolism in cells by increasing glucose and lipid absorption and activating the rate of oxidation to optimize cellular energy consumption (Monophosphate, 2017). Additionally, AMPK activation is believed to counter many cellular disturbances, such as inflammation, insulin resistance, and abnormal fat deposition (Ruderman et al., 2013). Crosstalk between AMPK and NLRP3 inflammasome signaling is thought to exist. NLRP3 activity is decreased by AMPK activation (Yang et al., 2019).

Converging evidence from research in human patients and corresponding animal models of the disease suggests that the proinflammatory cytokine interleukin 6 (IL6) plays a role in the pathophysiology of depression (Mohamed et al., 2018; Hammad et al., 2021). The most widely prescribed antidepressant medications, selective serotonin reuptake inhibitors, or SSRIs, primarily act through the serotonin transporter (SERT, SLC6A4). SERT activity shapes serotonergic neurotransmission, which is linked to the pathophysiology and behavioral traits of depression (Blier and El Mansari, 2013; Thorne, 2023). The binding of IL-6 to its receptor (IL-6R) commences the IL-6 trans-signaling mechanism in MDD. The resultant IL-6/IL-6R complex interacts with the gp130 component (Ting et al., 2020). When gp130 interacts with this complex, it activates Janus kinases (JAK) and the signal transducer and activator of transcription factor 3 (STAT-3), a downstream effector signal (Guan et al., 2021). STAT-3 was discovered to be increased in MDD patients (Guan et al., 2021). The inhibition of IL-6-stimulated STAT3 activation reduced the severity of depressive-like behavior (Kong et al., 2015). These findings emphasize the role of IL-6 and STAT3 molecular signaling in the evolution of MDD.

The monoaminergic, cholinergic, and glutamatergic systems, which have been linked to a number of neuropsychiatric illnesses, are all affected by statins in a broad range of neurotransmission processes. Such changes in neurotransmitter levels can be explained by both cholesterol-dependent and independent (such as anti-inflammatory and antioxidant) mechanisms (Giorgi Riccardo et al., 2023). Peroxisome proliferator-activated receptor (PPAR) is a receptor that is liganded by statins and regulates the expression of neurotrophins such as brain-derived neurotrophic factor (BDNF) (Giorgi Riccardo et al., 2023). Drug repositioning, or drug repurposing, is an effective strategy to find new indications for existing drugs. This strategy has been used with success across multiple diseases. Numerous studies support the idea that taking statins may have a favorable effect on mood, and several preclinical studies investigated the neuroprotective effect of statins on depressive behavior and reduced inflammation among MDD patients (Taniguti et al., 2019; Hai-Na et al., 2020; Massardo et al., 2022; Xiao et al., 2023). Haghighi et al. investigated the role of atorvastatin in alleviating MDD symptoms, but they did not measure any serum biomarkers (Haghighi et al., 2014). Therefore, the current research aimed at discovering the mechanistic role of atorvastatin in patients with MDD by modulation of AMPK/NLRP3 and IL-6/STAT-3 Signaling Pathways.

## 2 Patients and methods

From June 2023 to December 2023, the research was conducted at Tanta University’s Faculty of Medicine’s Psychiatry Department. The outpatient clinic recruited 60 eligible participants who fulfilled the inclusion criteria for the study. The Institutional Review Board at Tanta University Faculty of Medicine gave its acceptance for this work with the approval code (36264PR197/5/23). The study’s methodology and design adhered to the Helsinki Declaration and its 1964 revisions. Patients were told they could withdraw from the trial at any moment. The exposure type and randomization were blinded by patients and doctors. An unblinded pharmacist provided study drugs to participants to guarantee accurate treatment assignment; however, the pharmacist was not included in outcome evaluations.

### 2.1 Inclusion criteria

HAM-D score >18, with item 1 (depressed mood) scored two or above, and patients aged ≥18 years with a diagnosis of MDD based on the DSM-IV Mini-International Neuropsychiatric Interview (MINI) (Sheehan et al., 1998; Svenaeus, 2013) were eligible for the study. This study included only newly diagnosed cases of MDD.

### 2.2 Exclusion criteria

Patients suffering from personality disorders, eating disorders, drug abuse, or bipolar I or II disorders.

Patients having an active medical condition concurrently.

Patients who have previously experienced seizures or are undergoing electroconvulsive therapy (ECT).

Patients with known drug allergies or other contraindications.

Pregnant or lactating females.

Patients with hyperlipidemia and/or statin usage (defined as TC more than 200 mg/dL, or LDL more than 140 mg/dL, or TG more than 150 mg/dL).

Patients with diabetes, and hyperthyroidism.

### 2.3 Study design

**FIGURE 1:**
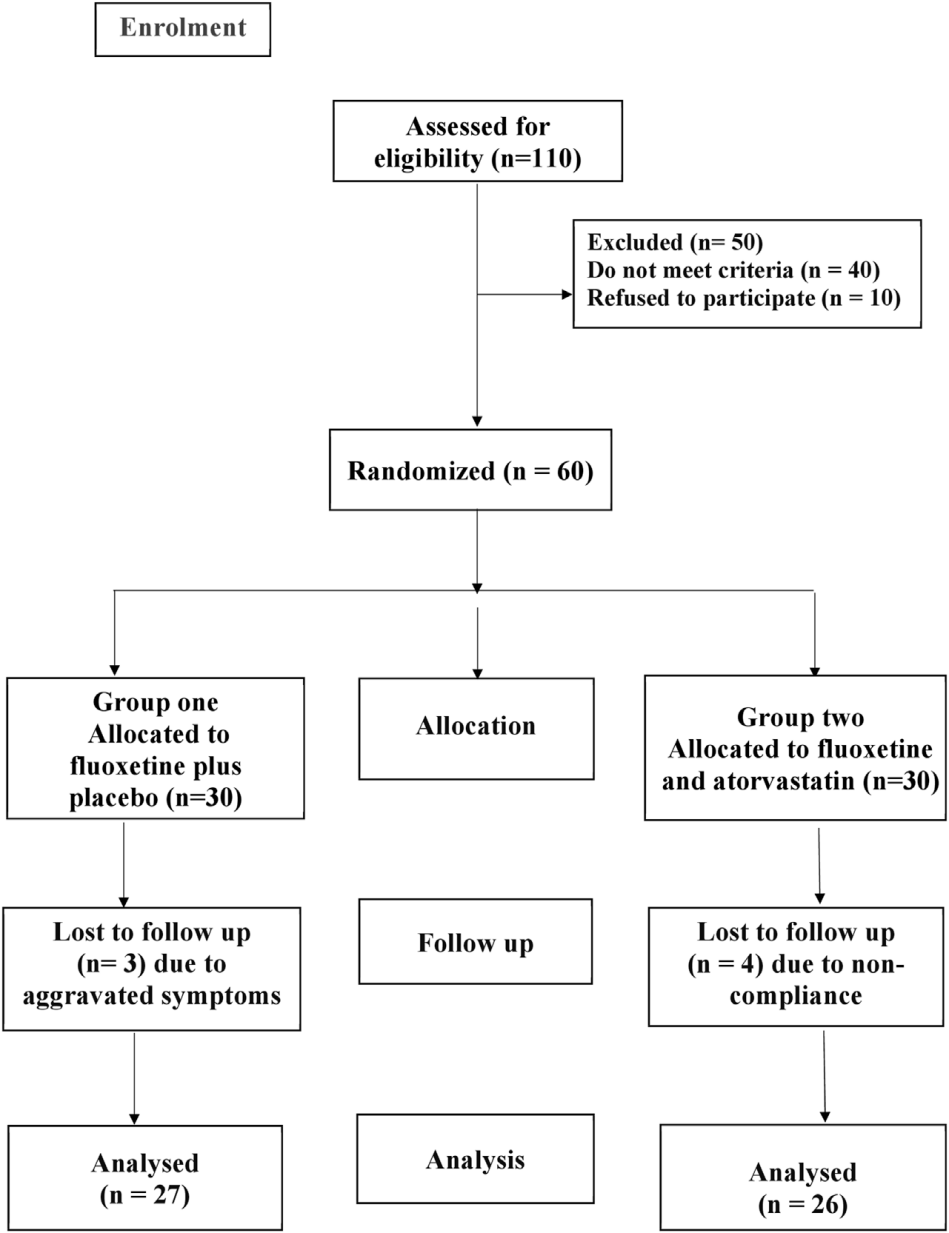
CONSORT diagram showing flow chart of the patient participation.

This clinical trial was prospective, parallel, randomized, and double-blinded. ClinicalTrials.gov received this trial’s registration as NCT05792540 in March 2023. This study was planned with three arms and registered on clinicaltrials.gov. Here we have data for only two arms. The participants were randomly divided into two groups (n = 30), as shown by the CONSORT flow diagram in Figure 1.

Atorvastatin dose selection was based on a network meta-analysis that recommended high-intensity atorvastatin in a high dose as the best choice for treating depression (Sparks et al., 2005; Lee et al., 2021). The patients were monitored for any adverse effects, including muscle pain, joint pain, or any other adverse effects. Blood-borne markers of liver dysfunction were also monitored as indicators of possible adverse events known to accompany the administration of atorvastatin 80. In addition, the fixed dose was chosen to preserve external validity, prevent blinding, and lessen the possibility of post-randomization biases.

Randomly permuted blocks were chosen for randomization using a computer random number generator. Before taking part in the study, patients had to be off all anti-inflammatory and psychiatric drugs that were used on demand, such as anxiolytics, hypnotics, and psychotropic drugs, for at least 4 weeks. Group 1: control group (n = 30) who received fluoxetine (20 mg) plus placebo once daily for 3 months (Philozac^®^20 mg capsule, Amoun, Egypt). Group 2: atorvastatin group (n = 30) who received fluoxetine (20 mg) once daily plus atorvastatin 80 mg once daily for 3 months (Ator^®^ 80 mg tablets, Epico, Egypt). Placebo tablets were manufactured by Zeta Pharma Company and had the same look as atorvastatin tablets.

### 2.4 Sample size calculation

Based on HDRS data as the primary dependent variable from prior trials using statin adjuvant therapies in MDD (Ghanizadeh and Hedayati, 2013; Haghighi et al., 2014; Gougol et al., 2015), with an average effect size of 0.8, a type I error of 0.05, and a study power of 80%. The sample size was calculated to be 30 patients per group, taking into consideration a 20% dropout rate.

### 2.5 Study protocol

A psychiatrist evaluated the patients at baseline and 3 months after they received the medication. Patients were also questioned regarding drug adherence and potential adverse effects. Patients were contacted every week by phone to check on their compliance with the study drug, any negative side effects, and any indications of infection or inflammation. All medications were taken orally. To assess patient adherence to therapy, the quantity of tablets left in each supply delivered to the patients was counted. Only in cases of an emergency that called for knowledge of the current course of therapy should the responsible psychiatrist break the blinding. The participant would be removed from the trial once the blinding was broken. If a participant stopped taking the study drug for 7 days in a row, they were also removed from the research.

### 2.6 Sample collection

10 mL of venous blood was taken from the antecubital vein before the trial began and 3 months after the intervention. To centrifuge the blood for 10 minutes at a speed of 4500 g (Hettich Zentrifugen EBA 20), the blood was slowly placed into plain test tubes, allowed to coagulate, and then centrifuged. One portion of the serum was used for standard hepatic and renal function testing, and the other portion was kept frozen at −80 °C for cytokine analysis.

### 2.7 Biochemical analysis

Using a spectrophotometric kinetic technique, the liver enzymes aspartate transaminase (AST) and alanine aminotransferase (ALT) were measured. Serum creatinine levels, a marker of renal function, were measured using the Jaffé reaction. Using the manufacturer’s instructions (Sunredio, Shanghai), commercially available enzyme-linked immunosorbent assay (ELISA) kits were used to measure serum levels of STAT-3 (catalog no. 201-12-0651), IL-6 (catalog no. 201-12-0091), NLRP-3 (catalog no. 201-12-5748), and AMPK (catalog no. 201-12-0747).

### 2.8 Outcomes assessment

The 17-item HDRS was the main outcome measure. A HDRS total score of ≤7 indicates remission, partial remission [HDRS<17 and>7], and no remission [HDRS>18]. Response was defined as ≥ 50% drop in the HDRS total score. Serum levels of IL-6, STAT-3, NLRP-3, and AMPK were assessed at baseline and 3 months later as a secondary end measure to assess the treatment’s biological effects.

### 2.9 Statistical analysis

GraphPad Prism v9 (GraphPad Software, Inc., San Diego, CA, USA), a statistical analysis program, was used for the analyses. The normal distribution of continuous variables has been analyzed using the Shapiro-Wilk test. Significant differences within the group before and after therapy were determined using paired Student's t-tests. To find significant variations between groups before and after therapy, unpaired Student's t-tests were performed. In terms of numbers, qualitative variables were provided, while quantitative values were expressed as mean and SD. Using Pearson’s correlation coefficient, parameters were correlated. On categorical data, the Chi-square test or Fisher’s exact test was applied, as appropriate. All *p* values were two-tailed, with *p* < 0.05 regarded as statistically significant.

## 3 Results

### 3.1 Clinical and demographic characteristics

Regarding the demographic baseline data, there were no statistically significant differences between the control and atorvastatin groups as follows: age (*p* = 0.264, t = 1.127, df = 58), sex (*p* = 0.438, χ^2^ = 0.6007), weight (*p* = 0.868, t = 0.1669, df = 58), height (*p* = 0.542, t = 0.6132, df = 58), ALT (*p* = 0.211, t = 1.263, df = 58), BMI (*p* = 0.471, t = 0.7244, df = 58), AST (*p* = 0.209, t = 1.270, df = 58), and SrCr (*p* = 0.609) (Table 1). After 2 weeks of starting the trial, four patients were withdrawn from the atorvastatin group due to non-compliance with the study medications. Three patients were withdrawn from the control group because they developed severe depressive episodes and shifted to combined therapy with antidepressants. Fifty-three patients completed the study; accordingly, the statistical analysis was performed per protocol analysis for all measured parameters except for HDRS, which was performed per protocol analysis and intention to treat analysis. A protocol analysis was used to assess the treatment’s biological and causative effects.

**TABLE 1:** Clinical and demographic data in the two study groups.

Parameter	Group (1) control group (n = 30)	Group (2) atorvastatin group (n = 30)	*p*-value
Age (year)	33.43 ± 5.93	35.13 ± 5.75	0.264 (t = 1.127, df = 58)
Sex (M/F)	17/13	14/16	0.438 (χ^2^ = 0.6007)
Height (m^2^)	1.661 ± 0.077	1.650 ± 0.064	0.542 (t = 0.6132, df = 58)
Weight (kg)	65.27 ± 5.607	65.03 ± 5.216	0.868 (t = 0.1669, df = 58
BMI (Kg/m^2^)	23.65 ± 1.33	23.87 ± 0.957	0.471 (t = 0.7244, df = 58)
ALT (U/L)	25.97 ± 4.664	27.47 ± 4.531	0.211 (t = 1.263, df = 58)
AST (U/L)	29.43 ± 4.840	27.93 ± 4.291	0.209 (t = 1.270, df = 58)
SrCr (mg/dL)	0.946 ± 0.134	0.963 ± 0.116	0.609 (t = 0.5132, df = 58)
Moderate depression	12 (40%)	10 (33.33%)	0.715 (χ^2^ = 0.133)
Severe depression	18 (60%)	20 (66.66%)	0.799 (χ^2^ = 0.064)

Data are expressed as mean ± SD, numbers, and percentage, M: male, F: female, ALT: Alanine amino-transferase, AST: Aspartate amino-transferase, SrCr: Serum creatinine, BMI: body mass index, Significance at (*p* < 0.05).

### 3.2 Effect of study medications on Hamilton Depression Rating Scale

Table 2 demonstrated no significant changes in baseline values between the two study groups using an unpaired t-test (*p* > 0.05). Compared with the baseline data, a paired t-test showed that there was a significant decrease in HDRS total score in the placebo group (25.33 ± 1.90 *versus* 15.33 ± 2.370, *p* > 0.0001, t = 15.07, df = 26) and the atorvastatin group (25.43 ± 1.794 *versus* 12.85 ± 2.11, *p* > 0.0001, t = 40.97, df = 25). The atorvastatin group showed a statistically significant decrease in HDRS total score compared with the control group after treatment [(*p* = 0.0002, t = 4.959, df = 51) and (*p* = 0.04, t = 2.052, df = 58)] per protocol analysis and intention to treat analysis, respectively. The response rate was 73.07% (n = 19/26) in the atorvastatin group *versus* 44.44% (n = 12/27) in the placebo group (*p* = 0.03, χ^2^ = 4.472).

**TABLE 2:** Comparison of Hamilton depression rating score and serum biomarkers in the two study groups.

Character	Group (1) control group (n = 27)			Group (2) atorvastatin group (n = 26)			^*b*^ *p*-value
	Before treatment	After treatment	^a^ *p*-value	Before treatment	After treatment	^a^ *p*-value	After treatment
HDRS (ITT)	25.33 ± 1.90	16.67 ± 3.754	>0.0001 (t = 10.20, df = 29)	25.43 ± 1.794	14.30 ± 5.08	>0.0001 (t = 12.95, df = 29)	0.04 (t = 2.052, df = 58)
HDRS (PPA)	25.33 ± 1.90	15.78 ± 2.722	>0.0001 (t = 15.07, df = 26)	25.43 ± 1.794	12.50 ± 2.025	>0.0001 (t = 40.97, df = 25)	0.0002 (t = 4.959, df = 51)
IL-6 (pg/mL)	191.4 ± 19.1	186.1 ± 20.34	0.016 (t = 2.568, df = 26)	193.3 ± 17.89	173.5 ± 17.14	0.0006 (t = 3.849, df = 25)	0.018 (t = 2.429, df = 51)
STAT-3 (pg/mL)	279 ± 11.44	222.4 ± 7.623	>0.0001 (t = 53.79, df = 26)	277.2 ± 10.57	179.4 ± 36.19	>0.0001 (t = 12.55, df = 25)	>0.0001 (t = 6.042, df = 51)
NLRP-3 (pg/mL)	164.3 ± 12.43	156.6 ± 12.19	0.002	162.7 ± 11.71 (t = 3.412, df = 26)	147.0 ± 10.84	>0.0001 (t = 6.215, df = 25)	0.004 (t = 2.998, df = 51)
AMPK (ng/mL)	65.81 ± 5.135	146.1 ± 15.06	>0.0001 (t = 28.26, df = 26)	64.29 ± 3.255	157.1 ± 15.85	>0.0001 (t = 31.27, df = 25)	0.015 (t = 2.574, df = 51)

Data are expressed as mean ± SD, Significance at (*p* < 0.05). per protocol analysis (PPA), intension to treat analysis (ITT), Interleukin-6 (IL-6), adenosine monophosphate activated protein kinase (AMPK), nucleotide-binding domain, leucine-rich–containing family, pyrin domain–containing-3 (NLRP-3), signal transducer and activator of transcription factor-3 (STAT-3), Hamilton Depression Rating Scale (HDRS), (a) within group comparison, (b) between group comparison.

No patient achieved full remission in both groups. Partial remission was (n = 24/26, 92.3%) for the atorvastatin group *versus* (n = 16/27, 59.25%) for the placebo group (*p* = 0.005, χ^2^ = 7.815).

### 3.3 Analysis of serum biomarkers

An unpaired t-test revealed no discernible differences in baseline values between the two groups (*p* > 0.05). In comparison to the control group, the atorvastatin group had a significantly elevated level of AMPK (*p* = 0.015, t = 2.574, df = 51) and a statistically significant decrease in the levels of IL-6 (*p* = 0.018, t = 2.429, df = 51), NLRP-3 (*p* = 0.004, t = 2.998, df = 51), and STAT-3 (*p* > 0.0001, t = 6.042, df = 51) following therapy. Furthermore, after medication, both groups’ serum levels of NLRP-3, STAT-3, and IL-6 reduced significantly from their initial values. However, when compared to baseline values, Table 2 shows a statistically significant increase in AMPK serum levels after medication in both groups.

### 3.4 Correlation analysis between measured parameters

There was a significant positive correlation between HDRS and NLRP3 (r = 0.431, *p* > 0.0001), HDRS and IL-6 (r = 0.3, *p* = 0.002), HDRS and STAT3 (r = 0.841, *p* > 0.0001), and AMPK and STAT3 (r = 0.8, *p* > 0.0001). On the contrary, there was a significant negative correlation between HDRS and AMPK (r = −0.918, *p* > 0.0001), IL-6 and STAT3 (r = −0.296, *p* = 0.002), and AMPK and NLRP3 (r = −0.386, *p* = 0.001).

### 3.5 Analysis of lipid profile in the two study groups

No significant changes in baseline values were recorded between the two study groups using an unpaired t-test (*p* > 0.05) (Table 3). After treatment, the atorvastatin group showed a statistically significant reduction in the levels of TC (*p* = 0.037, t = 2.137, df = 51), TG (*p* = 0.030, t = 2.227, df = 51), LDL (*p* = 0.007, t = 2.812, df = 51), and a statistically significant increase in HDL (*p* = 0.019, t = 2.403, df = 51) when compared to the control group. Additionally, after therapy, serum levels of TC, LDL, and TG in the atorvastatin group decreased statistically, and serum levels of HDL increased significantly when compared to their baseline values. On the other hand, Table 2 demonstrates no significant changes in serum levels of all lipid markers following therapy in the fluoxetine group when compared to baseline values.

**TABLE 3:** Analysis of lipid profile in the two study groups.

Character	Group (1) control group (n = 27)			Group (2) atorvastatin group (n = 26)			^*b*^ *p*-value
	Before treatment	After treatment	^a^ *p*-value	Before treatment	After treatment	^a^ *p*-value	After treatment
TC (mg/dL)	162.8 ± 16.94	164.7 ± 14.17	0.276 (t = 1.111, df = 26)	163.1 ± 17	156.3 ± 14.43	0.0003 (t = 4.161, df = 25)	0.037 (t = 2.137, df = 51)
TG (mg/dL)	135.1 ± 15.34	132.3 ± 10.28	0.311 (t = 1.033, df = 26)	128.3 ± 12.2	124.8 ± 13.87	0.013 (t = 2.670, df = 25)	0.030 (t = 2.227, df = 51)
HDL (mg/dL)	45.19 ± 8.13	43.85 ± 6.65	0.110 (t = 1.651, df = 26)	45.23 ± 8.83	48.42 ± 7.19	0.0004 (t = 4.049, df = 25)	0.019 (t = 2.403, df = 51)
LDL (mg/dL)	90.61 ± 17.78	94.43 ± 14.87	0.069 (t = 1.896, df = 26)	92.88 ± 18.74	82.95 ± 14.84	>0.0001 (t = 5.164, df = 25)	0.007 (t = 2.812, df = 51)

Data are expressed as mean ± SD, Significance at (*p* < 0.05). Total cholesterol (TC), triglycerides (TG), high density lipoprotein (HDL), low density lipoprotein (LDL), (a) within group comparison, (b) between group comparison.

### 3.6 Analysis of drug-related adverse effects between the groups

Table 4 showed that there were no significant differences between the studied groups in terms of side effects as followed: nausea (*p* = 0.480, χ^2^ = 0.497), stuffy nose (*p* = 0.111), fatigue (*p* = 0.300, χ^2^ = 1.074), muscle pain (*p* = 0.111), sexual dysfunction (*p* = 0.704), loss of appetite (*p* = 0.464, χ^2^ = 0.534), and insomnia (*p* = 0.690, χ^2^ = 0.158)**.**

**TABLE 4:** Comparison of drug-related adverse effects between the groups.

Side effect	Group (1) control group (n = 27)	Group (2) atorvastatin group (n = 26)	*p*-Value
Nausea	6	8	0.480 (χ^2^ = 0.497)
Stuffy nose	0	3	0.111
Fatigue	5	8	0.300 (χ^2^ = 1.074)
Muscle pain	0	3	0.111
Insomnia	6	7	0.690 (χ^2^ = 0.158)
Sexual dysfunction	3	4	0.704
Loss of appetite	5	7	0.464 (χ^2^ = 0.534)

Data were presented as numbers. Significance at (*p* < 0.05) using fisher exact test and chi-square test as appropriate.

## 4 Discussion

Although some clinical studies were conducted to assess the effect of atorvastatin, lovastatin, and simvastatin as adjunctive treatments in major depressive disorders, none of them measured any biomarkers that were related to inflammatory pathways in depression (Haghighi et al., 2014; Gougol et al., 2015; Massardo et al., 2022). To our knowledge, this is the first clinical research to assess the anti-inflammatory effect of high-dose atorvastatin in major depressive disorders through AMPK/NLRP3 and IL-6/STAT-3 signaling pathways. The safety of high-dose atorvastatin has been evaluated in patients for periods ranging from 2 weeks to 5 years, and rates of clinically significant myopathy and elevated hepatic enzymes were extremely low (Waters, 2005). The frequency and characteristics of adverse effects were anticipated and could be linked to the medications administered.

The current study demonstrated that atorvastatin treatment in combination with fluoxetine significantly reduced HDRS total score and produced a higher response rate than fluoxetine alone. This was demonstrated by a decrease in depression symptoms, as indicated by the HDRS score. As a result, this study supports the notion that atorvastatin could be an effective adjuvant treatment for MDD. This finding is consistent with the results of a previous study by Haghighi M. et al., which showed that atorvastatin potentiated the efficacy of citalopram (Haghighi et al., 2014). The higher dose of atorvastatin in our trial may explain the superior response and partial remission compared to Haghighi M. et al. Since it was reported that high-intensity atorvastatin at a high dose may enhance its antidepressant effects (Lee et al., 2021). Furthermore, our findings were consistent with previous research on the use of statins as adjuvant treatments for MDD, which showed that lovastatin and simvastatin had an antidepressant effect that increased the effectiveness of fluoxetine (Ghanizadeh and Hedayati, 2013; Gougol et al., 2015).

The current investigation showed that the atorvastatin group had significantly lower serum levels of IL-6 and STAT-3. These findings are consistent with and associated with earlier research in the same field (El-Mahdy et al., 2021). As STAT3 regulates IL6-dependent modulation of serotonin transporter activity and depressive-like behavior, targeting the IL-6/STAT-3 axis becomes a crucial approach in treating depressive-like behavior (Kong et al., 2015). Drug-induced STAT3 inhibition decreased depressive-like behavior and increased serotonin expression (Kong et al., 2015). According to studies, atorvastatin inhibits the IL-6/STAT3/endothelin-1 pathway in patients with spontaneous hypertension (Fang et al., 2019). Also, atorvastatin suppressed the IL-6/STAT3 pathway, resulting in the downregulation of human telomerase reverse transcriptase, which in turn causes atorvastatin-induced senescence of inflammation (Wang et al., 2020). In accordance with our study, celecoxib, an anti-inflammatory drug, exhibited antidepressant activity by suppressing serum IL-6 and reducing HDRS when compared to the placebo group (Gędek et al., 2023). The authors also found a significant correlation between a reduction in HDRS and a reduction in serum IL-6 levels (Gędek et al., 2023). These observations support the idea that atorvastatin could modulate depressive behaviors by modulating the IL-6/STAT-3 axis.

Furthermore, our study demonstrated a significant reduction in serum levels of NLRP3 and a significant increase in AMPK in the atorvastatin group compared to the baseline and fluoxetine group. These results are matched and in accordance with other reports in the same field (Wang et al., 2017; Yang et al., 2019). It is well known that NLRP3 contributes to the chance of acquiring MDD in several studies (Alcocer-Gómez et al., 2014; Pandey et al., 2021; Han et al., 2022). The methylation scores of cg18793688 and cg09418290 in the NLRP3 domain were substantially associated with cortical thickness in MDD patients (Han et al., 2022). Thus, targeting the NLRP3 axis could be a therapeutic strategy for treating MDD. The inhibitory activity of atorvastatin on NLRP3 and the activation of AMPK were explained by several mechanisms. Atorvastatin inhibits NLRP3 inflammasome activation via TLR4/MyD88/NF-κB signaling (Kong et al., 2016). Statins inhibit pregnane X receptor (PXR)-dependent NLRP3 inflammasome activation in vascular endothelial cells by oxidizing LDL cholesterol or TNFα (Wang et al., 2017). Statins also suppress NLRP3 by increasing the expression of aqua porin proteins (AQP2) (Kong et al., 2020) and promoting autophagy through the Akt/mTOR signaling pathway (Han et al., 2018). Atorvastatin also stimulates autophagy by stimulating AMPK production (Dehnavi et al., 2021) by directly activating the enzyme unc-51-like kinase 1 (ULK1) and blocking the mTORC1 complex’s inhibitory action on ULK1 (Mihaylova and Shaw, 2011).

Regarding the control group, fluoxetine showed a significant reduction in the levels of STAT-3, NLRP-3, and IL-6, along with a statistically significant elevation in the levels of AMPK compared to their baseline values. Certainly, fluoxetine’s role as an SSRI might be used to explain these results. Antidepressants may have a significant anti-inflammatory impact due to serotonin uptake inhibition (Ohgi et al., 2013). Moreover, fluoxetine can upregulate the serum neurotrophic factors and decrease inflammatory biomarkers in depressed patients, as reported previously (García et al., 2022). In depressed people, fluoxetine reduces levels of IL-6, IL-1β, and TNF-α in the pro-inflammatory pathway (García et al., 2022). In a prior study, fluoxetine therapy reversed an increase in TNF-α and a decrease in BDNF caused by lipopolysaccharides (LPS) in the hippocampus and prefrontal cortex (Taniguti et al., 2019). The effects of fluoxetine on interleukin levels, however, vary among studies. Some authors have noted a decline in serum levels of IL-6 (Lu et al., 2017), whereas others have documented no changes (Amitai et al., 2020). The therapeutic advantages of the combined therapy over the control group are therefore most likely attributable to high dose atorvastatin by altering the signaling pathways for AMP/NLRP3 and IL-6/STAT-3.

Regarding the statistically significant correlation between HDRS and measured parameters, reducing HDRS and relieving depression symptoms might lead to a reduction in IL-6, STAT3, NLRP3, and an elevation in AMPK. Also, there was a positive significant correlation between IL-6 and STAT3, and a negative significant correlation between NLRP3 and AMPK. It is well known that IL-6 is a potent activator and regulator of STAT3 signaling, and they are highly correlated (Guan et al., 2021). Furthermore, there was a significant positive correlation between AMPK and STAT-3. These findings are matched and correlated with previous studies (Mohamed et al., 2018; Taniguti et al., 2019). There is a crosstalk between AMPK and STAT3, as reported by several studies (Li et al., 2014; Speirs et al., 2018). AMPK and its activators lead to a reduction in STAT3 and its activity in various inflammations (Kim et al., 2020). In mouse liver and human hepatocytes, pharmacological stimulation of AMPK reduces the inflammatory reactions triggered by IL-6 and STAT3 signaling (Nerstedt et al., 2013). All of these observations might explain this correlation, and there was a crosstalk between the IL-6/STAT3 and AMPK/NLRP3 axes.

Our study demonstrated that the serum lipid profile was significantly improved in the atorvastatin group compared to the fluoxetine group and their baseline values. These results were in line with previous reports (Avisar et al., 2008; Jose et al., 2012). Atorvastatin efficacy in managing hyperlipidemia was widely investigated (Jose et al., 2012). The advantages of statins stem from their ability to decrease the biosynthesis of cholesterol, primarily in the liver, where they are specifically distributed. Additionally, they influence lipid metabolism through their inhibition of HMG-CoA reductase (Hu et al., 2021).

Inflammation is unlikely to be relevant for all patients with depression. However, it is established that mean concentrations of peripheral inflammatory markers are higher in depressed patients compared with controls (Osimo et al., 2020). Depressed patients are about 50% more likely to have evidence of inflammation as compared to matched non-depressed controls. Furthermore, it was reported that acute depression is a pro-inflammatory state, which lends support to the hypothesis that inflammatory marker elevations in depression are not due to an inflamed sub-group but rather to a right shift of the immune marker distribution (Osimo et al., 2019; Osimo et al., 2020).

Given that each drug is metabolized by a separate isoenzyme, it is expected that there were no pharmacokinetic interactions between atorvastatin and fluoxetine recorded (Mandrioli et al., 2006; Park et al., 2008). Additionally, there were no reported clinically important adverse effects and no notable variations in the clinical parameters of the patients, such as their age, gender, liver function, or renal function. The therapeutic advantages of the combined treatment are therefore most likely attributable to high-dose atorvastatin by altering the signaling pathways for AMP/NLRP3 and IL-6/STAT-3.

The present study had a number of limitations, including its short duration, its small sample size, and its use of specific atorvastatin dosages, despite its optimistic results. The Middle Eastern population is the exclusive focus of the current investigation. Therefore, the advantage seen in this trial should be confirmed in multicenter investigations and in other ethnic groups, especially in western countries where depression is expected to be more prevalent and may be different from a clinical standpoint. Furthermore, the research ethical committee denied using the placebo alone in patients with severe depression. Suicide attempts or suicidal ideation were not included in the secondary outcomes. In addition, there were no self-assessment scales or cognitive tests. Furthermore, creatine kinase (CK) should be monitored before and after treatment. Therefore, future trials may include a placebo and an atorvastatin-only group to assess the statin’s and placebo’s impact on depression severity.

## 5 Conclusion

This double-blinded, randomized trial led us to the conclusion that, in terms of decreasing inflammatory markers, atorvastatin combination therapy with fluoxetine is preferable to fluoxetine monotherapy in the treatment of major depressive disorder. By altering the signaling pathways for AMP/NLRP3 and IL-6/STAT-3, atorvastatin may reduce the levels of inflammatory biomarkers. While the results, along with previous research, hint at potential benefits, data on statin usage in MDD is still limited, and there is no specific data identifying which MDD patient subpopulation would gain most from this combination. Recommendations for such therapy should await further, larger, and well-designed studies to evaluate these effects.

## Data Availability

The raw data supporting the conclusion of this article will be made available by the authors, without undue reservation.
