# Corrigendum: Effect of a high dose atorvastatin as added-on therapy on symptoms and serum AMPK/NLRP3 inflammasome and IL-6/STAT3 axes in patients with major depressive disorder: randomized controlled clinical study

**DOI:** 10.3389/fphar.2024.1464358

**Published:** 2024-08-29

**Authors:** Khlood Mohammad Aldossary, Lashin Saad Ali, Mahmoud S. Abdallah, Mostafa M. Bahaa, Thanaa A. Elmasry, Eman I. Elberri, Fedaa A. Kotkata, Ramy M. El Sabaa, Yasmine M. Elmorsi, Mostafa M. Kamel, Walaa A. Negm, Aya Ibrahim Elberri, Amir O. Hamouda, Hayam Ali AlRasheed, Muhammed M. Salahuddin, Mohamed Yasser, Manal A. Hamouda

**Affiliations:** ^1^ Department of Pharmacy Practice, College of Pharmacy, Princess Nourah Bint Abdulrahman University, Riyadh, Saudi Arabia; ^2^ Department of Basic Medical Science, Faculty of Dentistry, Al-Ahliyya Amman University, Amman, Jordan; ^3^ Physiology Department, Faculty of Medicine, Mansoura University, Mansoura, Egypt; ^4^ Department of Clinical Pharmacy, Faculty of Pharmacy, University of Sadat City (USC), Sadat City, Menoufia, Egypt; ^5^ Department of PharmD, Faculty of Pharmacy, Jadara University, Irbid, Jordan; ^6^ Pharmacy Practice Department, Faculty of Pharmacy, Horus University, New Damietta, Egypt; ^7^ Pharmacology and Toxicology Department, Faculty of Pharmacy, Tanta University, Tanta, Al-Gharbia, Egypt; ^8^ Department of Clinical Pharmacy, Faculty of Pharmacy, Tanta University, Tanta, Al-Gharbia, Egypt; ^9^ Department of Clinical Pharmacy, Faculty of Pharmacy, Menoufia University, Shebin El-Kom, Menoufia, Egypt; ^10^ Psychiatry Department, Faculty of Medicine, Tanta University, Egypt; ^11^ Pharmacognosy Department, Faculty of Pharmacy, Tanta University, Tanta, Al-Gharbia, Egypt; ^12^ Genetic Engineering and Molecular Biology Division, Department of Zoology, Faculty of Science, Menoufia University, Shebin El-Kom, Menoufia, Egypt; ^13^ Department of Biochemistry and Pharmacology, Faculty of Pharmacy, Horus University, New Damietta, Egypt; ^14^ Department of Pharmaceutics, Faculty of Pharmacy, Port Said University, Port Said, Egypt; ^15^ Department of Pharmaceutics and Industrial Pharmacy, Faculty of Pharmacy, Horus University, New Damietta, Egypt

**Keywords:** atorvastatin, major depressive disorder, neuroinflammation, NLRP-3, STAT-3

In the published article, there was an error regarding the **Affiliation** for Manal A. Hamouda. Instead of having the affiliation “7 Pharmacology and Toxicology Department, Faculty of Pharmacy, Tanta University, Tanta, Al-Gharbia, Egypt,” they should be affiliated with “9 Department of Clinical Pharmacy, Faculty of Pharmacy, Menoufia University, Shebin El-Kom, Menoufia, Egypt.”

The authors apologize for this error and state that this does not change the scientific conclusions of the article in any way. The original article has been updated.

